# Editorial: Animal models of vascular interventions

**DOI:** 10.3389/fcvm.2025.1672089

**Published:** 2025-08-07

**Authors:** Ilia Fishbein, David A. Tulis

**Affiliations:** ^1^ Department of Pediatrics, Division of Cardiology, The Children’s Hospital of Philadelphia, Philadelphia, PA, United States; ^2^ Department of Physiology, Brody School of Medicine, East Carolina University, Greenville, NC, United States

**Keywords:** bioreactor, grafts, preconditioning, restenosis, vascular, zinc-aspirin, revascularization, animal model

**Editorial on the Research Topic**
Animal models of vascular interventions

Percutaneous interventions including laser or balloon angioplasty, atherectomy, and/or endovascular stenting, remain the established treatment for vascular occlusion and tissue hypoperfusion; however, in some patients, restenosis can occur and present a significant clinical challenge. Preclinical animal models are essential for the development, evaluation, and advancing our understanding of the biological response(s) to vascular disease or injury, the pathophysiologic mechanisms of occlusive restenosis, thrombosis, and healing, and the safety and efficacy of novel and potentially promising approaches and devices needed prior to human trials.

In this Research Topic, we explore unique *in vivo* and *ex vivo* experimental models that could help streamline translation of preclinical research to clinical efficacy for restenosis prevention and treatment. While no animal model perfectly recapitulates the human condition, the strategic use of novel and sometimes complementary animal models can help bridge the gap between basic research and clinical application. Featured in this Topic, Benke et al. used a rat heterotopic aortic graft transplantation/revascularization model and compared protection by preconditioning with aspirin vs. that from zinc-aspirin. Considering the known protective capacities of zinc, the authors hypothesized that preconditioning vascular grafts with zinc-aspirin would enhance protection against endothelial damage, inflammation, and oxidative stress (compared to aspirin alone) post-transplant. Vascular contractility, inflammation and apoptosis, and vessel patency and graft survival were assessed. The authors observed that preconditioning with zinc-aspirin reduced endothelial damage and provided protection in arterial grafts and concluded that zinc-aspirin may enhance arterial graft survival, patency, and function following revascularization. Use of rodent models including the rat for analyses of vascular intervention and post-interventional revascularization is generally appropriate and cost-effective and allows mechanistic insight into injury, restenosis, and remodeling as well as preclinical discovery and characterization of potential new therapeutics.

A study by Riber et al. evaluated carotid-jugular arteriovenous (AV) graft patency and function in a sheep model and compared a novel Biomodics^©^ interpenetrating polymer network (IPN) drug-eluting graft to a traditional heparin-coated GORE® ACUSEAL graft. The authors aimed to determine if the Biomodics^©^ IPN grafts were functionally superior to ACUSEAL grafts in a preclinical AV revascularization model. Bilateral end-to-end AV conduits from the common carotid artery to the jugular vein in female sheep were created, and animals were evaluated using ultrasonic duplex scanning for graft patency over 12 months. Interestingly, materials in the IPN grafts were not stable *in vivo* and degraded over time, leading to vascular occlusion and graft failure. In some IPN grafts, a fibrotic sheath encapsulated the degraded material, contributing to vessel occlusion. While the ACUSEAL grafts were biocompatible and maintained structural integrity, they eventually failed due to exaggerated neointimal hyperplasia. In this study, while nearly every IPN and ACUSEAL graft occluded within the 12-month evaluation period, the authors felt that the IPN grafts warranted continued study as they lacked overt neointimal growth and could benefit from construction using more biocompatible materials. In these experiments, sheep represented a suitable model due to vessel size and hemodynamics comparable to those in humans, yet cost, ethical concerns, and regulatory constraints may limit their broad utility.

A common element of endovascular intervention in both experimental and clinical models is percutaneous access to the vasculature. Retrieval of the hemostatic sheath at the end of a procedure in an anti-coagulated subject causes bleeding that can be clinically significant and necessitate urgent surgical repair. Traditionally, this bleeding is controlled by 4–6 h of external mechanical pressure, which is cumbersome for patients and precludes early ambulation. Vascular closure devices (VCDs) are an alternative strategy for controlling post-procedural hemostasis. VCDs have gained clinical recognition over the past 20 years and are currently used in ∼60% of all femoral access procedures. In this Research Topic, Perkins and Tu reviewed the utility of diverse animal models for preclinical evaluation of VCDs. In addition to providing an updated classification of VCDs, the authors discussed the anatomical characteristics of common large animal models (porcine, ovine, caprine, and canine) as they relate to VCD implantation techniques. Special attention was paid to study design, which included the correct choice of controls and ensured reproducible data across multiple endpoints and duration times while minimizing the number of experimental animals. The authors also discussed methods of performance evaluation of VCDs such as time to hemostasis, lack of prolonged vasospasms, acute thrombosis, embolization, and both local and systemic reactions. Overall, this comprehensive review article presents a valuable guide for testing implantable VCDs for hemostasis control.

In addition to *in vivo* models, *ex vivo* preparations can offer several advantages such as precise control of experimental conditions and ease of access for intervention and/or observation. A study by Razzi et al. detailed use of ex vivo vascular bioreactors (VABIOs) under simulated flow and pressure conditions to assess efficacy of coronary artery stent implants. Using swine hearts surplused from slaughterhouse operations, these investigators validated VABIO as a viable alternative to *in vivo* and *in vitro* models for study of the vascular responses to biomechanical and biochemical factors associated with stent deployment. Swine coronary arteries closely mimic human arteries and are considered the gold standard for assessment of vascular stents; however, high cost and ethical concerns limit their broad use. This study helps validate the use of slaughterhouse swine hearts in VABIO as an established approach for thorough evaluation of vascular stents.

Lastly, a study by Belhoul-Fakir et al. described a novel porcine model of atherosclerotic plaque initiation and early evolution. Autologous blood microinjections into the tunica media of the infrarenal aorta combined with a 12-week high-fat diet resulted in localized lipid accumulation at the injection sites. The adjacent arterial segments remained spared from lipid accumulation. The lipid deposits co-localized with markers of vasa vasorum, neutrophils, macrophages, and T- and B-leukocytes, recapitulating the complex pathology of nascent atherosclerotic plaques. The authors leveraged their histological data to challenge the prevailing concept that atherosclerotic plaque is initiated in a dysfunctional intimal layer, arguing that injured media can also serve as a nidus for plaque development. While the role of intraplaque hemorrhage in the non-linear growth of atherosclerotic plaque is well-recognized, the iatrogenic modeling of vessel wall hemorrhage has not been previously used in atherosclerosis research.

In conclusion, the diverse experimental models detailed in this Research Topic provide critical insights into the biological responses to vascular intervention in the preclinical setting. Through judicious use of these and other animal models, the gap between basic science inquiry and clinical utility may be bridged.

